# An analysis of ophthalmology services in Finland - has the time come for a Public-Private Partnership?

**DOI:** 10.1186/1478-4505-7-24

**Published:** 2009-11-10

**Authors:** Liina-Kaisa Tynkkynen, Juhani Lehto

**Affiliations:** 1 University of Tampere, Tampere School of Public Health, 33014 University of Tampere, Tampere, Finland

## Abstract

**Background:**

We studied the prerequisites for Public-Private Partnership (PPP) in the context of the Finnish health care system and more specifically in the field of ophthalmology. PPP can be defined as a more or less permanent cooperation between public and private actors, through which the joint products or services are developed and in which the risks, costs and profits are shared.

The Finnish eye care services system is heterogeneous with several different providers and can be regarded as sub-optimal in terms of overall resource use. What is more, the public sector is suffering from a shortage of ophthalmologists, which further decreases its possibilities to meet the present needs. As ophthalmology has traditionally been a medical specialty with a substantial private sector involvement in service provision, PPP could be a feasible policy to be used in the field. We thus ask the following research question: Is there, and to what extent, an open window of opportunity for PPP?

**Methods:**

In addition to the previously published literature, the research data consisted of 17 thematic interviews with public and private experts in the field of ophthalmology. The analysis was conducted in two stages. First, a literature-based content analysis was used to explore the prerequisites for PPP. Second, Kingdon's (1995) multiple streams theory was used to study the opening of the window of opportunity for PPP.

**Results:**

Public and private parties reported similar problems in the current situation but defined them differently. Also, there is no consensus on policy alternatives. Public opinion seems to be somewhat uncertain as to the attitudes towards private service providers. The analysis thus showed that although there are prerequisites for PPP, the time has not yet come for a Public-Private Partnership.

**Conclusion:**

Should the window open fully, the emergence of policy entrepreneurs and an opportunity for a win-win situation between public and private organizations are required.

## Background

Since the emergence of the New Public Management (NPM) in the 1970s [1], redefining the boundaries between public and private sectors has drawn increasing interest. Along with the NPM, the public sector began to adopt a more market-oriented approach to arranging welfare services, and the view on the public sector as an irreplaceable actor in correcting the welfare differences and inequalities in society, was questioned. Among the policies that emerged as a consequence of the NPM was also the concept of Public-Private Partnership (PPP) [2]. The concept of PPP first appeared in the health care literature in the 1990s, and the term has gained popularity over the past decade [3]. In this article we define PPP as a more or less permanent cooperation between public and private actors, through which the joint products or services are developed and in which the risks, costs and profits are shared [2].

This study is situated in the context of the Finnish health care system and more specifically in that of ophthalmology, which is a part of specialized medical care in Finland. The Finnish health care system comprises three different levels, i.e. municipal health care, occupational health care and private health care, all of which receive public funding to some degree. Municipal health care is mainly funded through taxation, whereas private health care and occupational health care are funded by compulsory National Health Insurance (NHI) and by out-of-pocket payments. The municipalities (i.e. local authorities) are obliged by law to arrange primary and secondary care services for their citizens. Each municipality must belong to a hospital district, altogether 20 in Finland, that provides specialized health care for the population of their member municipalities[4] Furthermore, each hospital district belongs to one of the five university hospital responsibility areas that are accountable for providing the most specialized medical care, specialist training and research. In order to access public specialist medical care, i.e. public specialists and public hospitals, a referral from a licensed physician, either public or private, is needed [4]. No referral is needed to visit a private specialist.

As for the relationship between public and private sectors in Finnish health care, it can be said that the present situation is perhaps best characterized by the co-existence of the two sectors. While the private and public actors are operating in parallel, the sectors are not related as systems. Lately, some marginal cooperation between the two sectors has developed as the public sector has for instance purchased some surgical services from private enterprises. All in all there has not been, however, much room for partnership arrangements in the Finnish health care system. Hence, in most cases the public sector has been the dominant actor in terms of organizing, providing and funding health care services. However, there are a few fields where the private sector has traditionally played a major role, one of them being ophthalmology.

**Figure 1:**
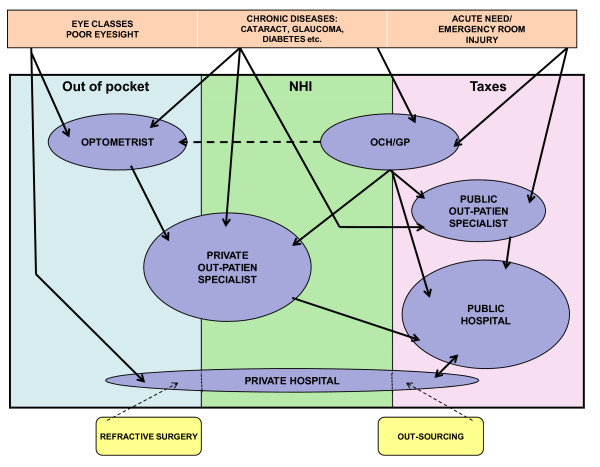
**Finnish eye care services system by funding channels and service providers**.

In Finland, ophthalmology has traditionally been a specialty in which the use and provision of private services have been more common than in health care on average. Other specialties with a relatively large share in private service provision in Finland are dental care [5] and gynecology [6]. Together with gynecology, ophthalmology accounted for over one-third of all private specialists visits in 2006 [4]. Moreover, as many as two out of three ophthalmology patients are currently managed by the private sector [7]. Eye care services are provided mainly by public and private specialists in outpatient clinics or hospitals and by optometrists in optical stores but also by general practitioners (GP) in occupational health care (OCH) and health centers, albeit it is rare for health centers to have ophthalmologists of their own. As a whole, the actors operating in the ophthalmology service system are multiple, and there are many different ways to access care (Figure 1).

The majority of Finnish ophthalmologists operate part-time within both the public and private sectors, which has contributed to the shortage of ophthalmologists in the public sector [8]. As the specialists' work is divided into two sectors, this kind of dual practice may lead to a wasteful use of health care resources. What is more, as far as service provision is concerned as a whole, the heterogeneous service system may take the aggregate resource allocation even more under the ideal level. Finally, the ageing of the population, new technologies and new forms of care further increase the challenges facing ophthalmology.

In this study we examine the prerequisites for Public-Private Partnership (PPP) in the context of Finnish ophthalmology services. As the private sector's share in the ophthalmology services is relatively considerable, we believe that PPP could be an adequate policy for solving the current problems discussed above. We adopted an organizational viewpoint, as is common within PPP theories. Ophthalmologists have traditionally been sole practitioners in Finland, usually having a contractual relationship with the optical stores. Recently, however, large chains of health care companies have gained ground in ophthalmology and a multitude of ophthalmologists is employed by them. Consequently, the decisions are no longer made at the level of single practitioners but higher up in the organizations. Hence, an organizational approach can be deemed reasonable.

## Methods

### Theory

In order to study the prerequisites for PPP, we formulated an analysis framework based on the theoretical and empirical literature on PPP. It is not clear what different authors eventually mean by PPP [9]. Consequently, the concept of PPP is not a fixed policy concept but an umbrella term covering a variety concepts [10,11]. It was possible, however, to identify certain factors common to different PPP arrangements. As we were interested in the preconditions of PPP, we identified the factors found to affect the formulation of PPP from the literature. The findings were classified into three categories labeled as "mutual disadvantage and mutual benefit", "mutual values and mutual relationship" and "the wider societal context". We define PPP as a policy concept that is related to a shared goal or a shared problem, which the actors cannot meet or solve alone (e.g. actors have complementary resources). On the other hand, the prerequisites are related to the compatibility of the actors' organizational values and cultures, and to the context in which the partnership is planned.

In addition, PPP can be seen as an example of a policy change. As the changes in public policies take place through multiple processes affected by multiple actors and a wider societal context, we complemented the analysis with Kingdon's (1995) theoretical framework drawn from the social and political sciences [12]. The theory has been used also in previous studies on health care [13,14]. Kingdon (1995) argues that the current policies may change through three independent streams called the problem stream, the policy stream and the political stream. First, the change requires that the actors are able to find a common problem and are willing to solve it (an open window of opportunity in the problem stream). Second, to solve the problem, a feasible solution, of which sufficient mutual understanding prevails, must be found (an open window of opportunity in the policy stream). Finally, attention must be paid to the political atmosphere which dominates in society and to the environment in which the actors operate (an open window of opportunity in the political stream). According to the theory, a simultaneous opening of the window of opportunity in all the streams will make agenda change possible. In other words, the streams come together at critical times and when coupled together, a window of opportunity for agenda change will open [12].

We addressed the following research question: Is there, and to what extent, an open window of opportunity for PPP in ophthalmologic services in Finland?

### Data

The present study is part of a research project designed to explore new innovative ways to arrange ophthalmology services. At the beginning of the project in Summer 2007, a literature reviewed was conducted and altogether 17 experts were interviewed. Our informants represented the main public and private actors in ophthalmology in the responsibility area of Tampere University Hospital (TAUH). The group of private actors (n = 5) consisted of the representatives of three national chains of private health care, each of which has a substantial market share in ophthalmology services. The group of public sector actors consisted of the representatives of the responsibility area of TAUH. They came from public secondary health care, i.e. specialized medical care (n = 10), and from a large primary health centre (n = 1). The interview group consisted mainly of the management personnel of these organizations, but also included ophthalmologists (n = 4) and nursing staff (n = 1). The selection of the interviewees was based on identifying different viewpoints and ensuring saturation of the data.

Thematic interviews were conducted between Autumn 2007 and Spring 2008, and they were based on the interview frame developed by the researchers of the project. The purpose of the interviews was to explore the prerequisites of systemic health care innovations in health care, one of the aspects being the relationship between public and private sectors. Several themes were discussed in the interviews, e.g. the present problems of the ophthalmology service system, the current relationship between the public and private sectors and the actors' views on the possible new operational policies. The data used can be considered sufficient as it is compatible with the view of ophthalmology given by official documents and research literature.

In order to describe current public opinion concerning private service providers, we employed a study published by The Foundation for Municipal Development (FMD) in 2006. The study used a postal survey to explore the attitudes of citizens (n = 1 039) and municipal managers (n = 190) towards local government [15]. The study addressed two questions about the attitudes towards the involvement of the private sector in service provision. We used these questions to analyze current public opinion concerning the private sector.

### Analysis

The analysis was conducted in two stages using theory-based content analysis. We first analyzed whether the prerequisites for PPP were dealt with in the interviews. This was done by using the literature-based theoretical approach discussed above. The informants' factual statements were used as the unit of analysis. We did not aim at providing a comprehensive view of a single informant's way of thinking. Rather, we assumed and accepted that an interviewee may express even contradictory statements within a single interview. All the statements that discussed the relationship between public and private sectors, the problems in the current situation or possible policy proposals were understood as relevant for our analytical purpose. The statements were interpreted as views expressed by the actors in the policy arenas in question.

To conduct the second stage of the analysis we employed Kingdon's (1995) multiple streams theory of policy change. We aggregated the results from the first stage of the analysis following Kingdon's framework and drew on the study by FMD to examine whether there is an open window of opportunity for PPP in the field of ophthalmology.

## Results

### Analysis of the prerequisites for PPP

#### Mutual disadvantage and mutual benefit

Before starting the analysis we assumed that the current state of affairs appears disadvantageous for both the public and private service providers mainly due to the facts mentioned above. Furthermore, we assumed that both sectors could benefit from improvements in the present situation. Hence, we begin the analysis by examining whether the contemporary situation in the field of ophthalmology appears disadvantageous for the actors and whether future benefit could be gained with the help of PPP.

Awareness of the fact that the objectives set for an organization cannot be met alone may impede the initiation of PPP [9,16-18]. References to this were found in the data when the demand conditions were discussed. The actors of the public sector perceived the public sector's own resources to be inadequate with respect to demand, making it impossible to provide care to all patients in the current situation.

"The biggest problem at the moment is that the patient load has increased enormously and there is no chance that we could take care of them all" (Head Nurse, Pub)

The private actors, in turn, referred to problems that were mainly related to the perceived instability of demand. They felt that there was a lack of infrastructure and know-how needed to treat all the patients. The instable demand conditions, however, make it risky to acquire the devices and equipment necessary for the treatment of patients.

"Purchasing devices requires substantial monetary investments, but will the number of incoming patients cover the expenses?" (Ophthalmologist1, Priv)

Moreover, the private actors seemed to be afraid of the possible strengthening of the public sector, as the improvements in the public sector's scope of action would probably change the market position and the number of public sector service contracts. This, in turn, could make the competitive stance of private producers even more uncertain.

"The biggest specter here, in the private sector, is that the public sector is able to do all the things we do in the private side at the moment" (Manager1, Priv)

Recognition of the interdependency between public and private organizations was found to be another factor that may affect the initiation of PPP [9,16-18]. The issue emerged when discussing the division of labor.

"My conclusion was that we have a structure that perpetuates the shortage of ophthalmologists and the waiting lines. When the majority of ophthalmologists are working in both the public and private sectors, the system is a two-way street, which then creates the current structure." (Chief ophthalmologist1, Pub)

Thus the ophthalmologists' dual practice seems to cause a disadvantageous situation in terms of aggregate resource allocation. The current structure also seemed to blur the market conditions and cause conflicts of interest for individual practitioners. Finally, in addition to the dual practice, the specialists' monopoly on the supply of labor was said to increase health care costs, partly because specialists are in high demand.

"The cost of ophthalmology care has already risen in both sectors because the experts' charges are going through the roof" (Manager1, Priv)

The current situation appears to be disadvantageous especially from the point of view of the employers. The employees, i.e. ophthalmologists, for their part, are likely to regard the current situation as beneficial, as they possess strong negotiation power on the conditions of their work. Should the initiation of PPP succeed, it is crucial that the professionals working for the organizations are motivated to change the current situation [19]. Without internal legitimacy given to the formulation of PPP, there are no prerequisites for PPP [20]. According to our analysis, the possible change was considered both positive and negative by the ophthalmologists. However, resistance by the profession was mentioned frequently when a particular interest group possibly opposing PPP was named.

"The ophthalmologists are most probably the biggest single group of opponents"

(Manager1, Pub)

When multiple actors operate in the same field without a mutual agreement on the terms of cooperation, the division of labor and the responsibilities between the parties may appear unclear [19]. This may result in wasteful resource use and overlap in service supply. In the data, the vague roles in service provision were indeed seen suboptimal in aggregate.

"I'm totally convinced that more health could be produced if the use of the resources, currently allocated in ophthalmology, was better planned. Now the system is fragmented, divided into public and private and it isn't necessarily known what the private sector is doing. Our effectiveness falls short of optimal levels."

(Chief ophthalmologist1, Pub)

However, the PPP could increase the possibility for better resource allocation [17] and it could also be seen as a tool to understand a complex service system [21].

More effective resource allocation and service supply requires, however, that the actors are able to find clear roles in service provision [22]. It is also required that supplementary resources exist between the public and private sectors [16,23,24]. The distribution of labor was mentioned in the context of sight examinations and optical prescriptions, which were almost unanimously seen as tasks belonging to the private sector. Instead, more contradictory views between the sectors were connected to the management of cataract surgery:

"The university hospitals and the central hospitals should particularly invest in operations that cannot be carried out in the private sector."

(Ophthalmologist1, Priv)

A common private sector view was that the public sector should concentrate on the most difficult operations, specialist training and research, while routine operations, such as cataract surgery, could be undertaken by the private sector. The public sector actors did not share this view, as they wanted to retain the routine operations in the public sector. Both sides were unanimous in asserting that the most demanding tasks must be undertaken in the public sector, mainly because the private sector is lacking adequate equipment. At the same time, however, the refractive surgery procedures depend almost entirely on private supply, as they are not performed in public hospitals.

In the end, the formulation of PPP provides experience of its necessity and sensibility [23]. PPP could be a beneficial solution for the private sector because *"it would improve the profile value of the private sector in a totally different way" *as one of the interviewees described the matter. Demand in the public sector is fairly constant [25], partly due to the obligations arising from law, and the private sector might want to confirm its market position under uncertain demand conditions. In the public sector, in turn, the benefits were seen in the form of the technologies, and new types of services and practices that would diffuse from private to public sector if the PPP was formed.

"The line between the public and private sectors can possibly be crossed so that treating private patients in the public sector becomes possible. I would find it necessary. Effective practices from the private sector would be better integrated into public health care as they are in the same building anyway."

(Chief ophthalmologist2, Pub)

In addition, the public sector could acquire additional resources through the partnership and thus improve its capacity to provide services. However, we also identified negative attitudes, and it seemed that especially the public actors had a strong desire to operate independently without any external help.

All in all the attitudes towards the possible cooperation arrangements seemed to be contradictory. It may be that the need for PPP is realized but the ethos of the public sector talks against it. In the private sector the negative attitudes were mainly connected with the fact that the PPP was not considered a policy proposal capable of bringing any surplus value to the private organization. The discussion finally boils down to the values of the actors, which are discussed in the next section.

#### Mutual values and mutual relationship

The initiation of PPP may fail if the values and objectives of the parties differ considerably [18]. We found that the operating principles in the public and the private sectors were differently perceived.

"If we consider this clearly as a systemic matter, the private sector should be involved. However, the profit seeking interests of the private sector create a problem." (Manager2, Pub)

The quotation reflects a situation in which the profit seeking interests of the private sector seem to be clashing with the values of the public sector. In turn, private actors may be afraid that a PPP agreement between former competitors could endanger market competition [26]. This was brought up also by some of our informants. In addition, the political nature of public sector decision-making was found problematic by the private actors and this kind of obstacle to PPP has also been identified in the literature [27].

The values held by the specialist also direct the operations that are carried out within the sectors. The public and private sectors seem to offer different kinds of incentives for specialists. As one of the informants put it:

"Those who work for the private sector do it for money. In the public sector one can, in turn, best maintain ones professional skills." (Administrative nurse, Pub)

It seems that more demanding tasks make ophthalmologists willing to work for the public sector. One interviewee even reported that the possibility to operate was *"the spice of work"*. In addition, also the possibility to receive training must be included as an incentive to work for the public sector. By contrast, the private sector was considered a more pleasant working environment with its *"convenient working hours and comfortable posts" *as one of the interviewees reported.

The above-mentioned differences between the sectors also reverberate to differences between the patients treated and to the know-how needed in the public and private sectors [28].

"We have specific criteria for surgery and patients not meeting them are not operated on -- more ripe cataracts are sent here from the public sector but we have agreements to determine what is done here." (Manager2, Priv)

As this quotation shows, the present situation seems to make "cream skimming" possible for private actors. However, the public sector seems to practice similar kind of sub-optimizing, as it regards contracts with the private sector only as a last resort. This kind of "public sector cream skimming" as an obstacle to the PPP has been reported by other studies as well [10].

"Out of necessity, we have lately purchased a substantial amount of services from the private sector, but if we had an adequate capacity to render treatment, I don't see any reason to cooperate." (Manager2, Pub)

It was also evident that the private actors were mistrustful of the public sector as a service contractor. As one interviewee commented:

"In extreme cases of distress the cavalry is called in, but otherwise people are left to fend for themselves. The university hospital will not sign a contract until it is forced to render treatment" (Manager3, Priv)

Thus it is possible to conclude that while both sectors are willing to undertake only the operations optimal for them their activities are also underpinned by different values.

These differences comprise neither a constraint on nor an impetus for PPP per se. Rather, the way the differences are identified and taken into consideration is important [18]. This finally boils down to the good mutual relationship and confidence between the parties, which, when lacking, may impose potential constraints on any kind of relationship [20]. The analysis suggests that the relationship between the public and private sectors cannot be described as good. In the public sector the comments were associated with more general ideas about the private sector, whereas the private sector informants reported their own experiences from the field in more detail.

"If I have to send a patient with a complication to TAUH, I find it embarrassing. When that patient goes there they will ask if that private sector sad sack with huge earnings has again taken care of the business." (Ophthalmologist1, Priv)

In addition, the lack of mutual appreciation also emerged from the interviews. This is evidenced by the previous quotation, as well as by the fact that in the private sector it was felt that the communication between the sectors was not working. This has been identified as an obstacle to PPP in the literature as well [28]. While it seems that there are communication problems between the organizations, many of our informants stressed that the ophthalmologists in both sectors are part of a rather cohesive professional community with much lower barriers to communication.

Finally, certain public sector tasks and responsibilities, imposed by law, possibly impede the formulation of PPP [29]. The public sector actors may be afraid that, because of PPP, it may not be possible to fulfill all the public duties, e.g. training and research [18,30]. Also the questions of equal and sufficient supply of services, the efficient use of resources, the social responsibility and the safety of services may come up when the PPP is considered [9,16]. Some public sector informants also referred to social responsibility. In addition, the fear of endangering the specialist training and research, which mainly belong to the public sector, emerged in the interviews.

"How the research and training could be included bothers me" (Ophthalmologist1, Pub)

Finally, the political nature of the public sector's decision-making may be problematic from the private sector's point of view [27]; this was also what our analysis showed.

#### Wider societal context

The discussion about the possibilities of PPP must be considered inherently political, as the PPP is, in the end, a matter of allocation and redistribution of the scarce societal resources [31]. The public sector policy makers are dependent, at least in theory, on public opinion, and in order to consider the prerequisites for PPP, it is important to analyze whether public opinion supports private sector involvement in service provision [19]. To estimate public opinion on enhancing the role of the private service producers, we drew on the study conducted by The Foundation for Municipal Development (FMD 2006) (Table 1).

**Table 1:** Attitudes of citizens and local authority executive directors towards private service providers

	**Outsourcing of municipal services would increase inequality and insecurity among citizens (%)**				
	**Agree**	**Somewhat agree**	**Cannot say**	**Somewhat disagree**	**Disagree**

**Citizens**	23	43	4	26	3

**Manager**	2	32	4	44	17

	**Outsourcing of municipal services would result in better services and cost-savings (%)**				

	**Agree**	**Somewhat agree**	**Cannot say**	**Somewhat disagree**	**Disagree**

**Citizens**	7	41	5	33	14

**Manager**	12	46	5	32	4

Source: FMD 2006: 16-23, 62-64, 66-67, 69

Citizens' attitudes towards private service providers were fairly negative. An examination of the trend from the year 1990 to 2006 showed that the attitudes have grown increasingly negative over the past one and a half decades [15]. The municipal managers' opinions were less skeptical, but the increasing involvement of the private sector did not gain full support from them either. In both groups, most respondents reported that they "somewhat agreed/disagreed" with the statements of the study. Thus it seems that, in the end, public opinion on the matter remains uncertain.

The health care system must be regarded as part of a wider system, which determines the practices that are allowed in the health sector [32]. The acts and statutes resulting from the political process must be taken into account when PPP is planned, as legislation may forbid the formation of a partnership. The legislative constraint may emerge especially if changes to current legislation must be made. [9,22] References to the legislative constraints on the PPP also emerged from the data.

"What about legislation and health insurance fees? And whose premises will be used? And what about the charges; when will the hospital charges be used and when those of the private practices?" (Administrative nurse, Pub)

Under current Finnish legislation, it is not possible to execute all the forms of PPP, as the health insurance act rules out the reimbursement of private services in public premises [33]. There are, however, examples of arrangements that make it possible to bypass the legislation [4].

### Interpretation: how open is the window?

#### Problem stream

We define "a problem" as a state of affairs which is in conflict with the actors' appreciations and attitudes and to which a change is hoped for [12]. Thus the problem is not objectively determined but a question of the actors' subjective interpretations of the situation. In the analysis above we identified several problems that were shared by both sectors. However, even if the problems were common in the end, they were defined and described differently by public and private actors (Table 2).

**Table 2:** Perceived problems in the public and private sectors

**PROBLEM**	**PUBLIC**	**PRIVATE**
**Demand**	Excessive growth in demand	Perceived uncertainty of demand

**Public sector position**	Inadequate resources	Possible strengthening of the public sector

**Division of labor**	Vague roles	Public sector wants to retain the low-risk surgeries as well

**Ophthalmologists'** **dual practice**	Sub-optimal resource use	Sub-optimal resource use

The first two problems seem to concern more clearly the public sector alone. The majority of public sector informants saw that the growth in demand had surpassed the existing resources that were considered inadequate. The matter was discussed both generally and in the context of TAUH. However, the situation was problematic also from the private sector's point of view. Some private sector informants referred to problems related to the perceived uncertainty of demand for private ophthalmology services. Some others described their concerns about the possible strengthening of the public sector, which could change the market positions, i.e. possibly create a public monopoly in service supply.

The latter two problems, instead, seemed to concern both sectors similarly. When the division of labor was discussed, the actors of the public sector expressed it in the form of vague roles in service provision. The private actors, by contrast, felt that the public sector's willingness to hold on to the less demanding operations was the main problem. Finally, the fourth problem was defined similarly by both sectors. The ophthalmologists' dual practice was seen as a structure resulting in sub-optimal resource use. In the public sector this was embodied especially in structures which led to a shortage of ophthalmologists. In the private sector the problem was more about the ophthalmologists' high charges that increase the cost of service supply. In both sectors the resource use was problematic especially from the point of view of the employers.

In the end the problem seems to be, however, as follows:

"It is one of those 'every man wants to have his own thresher' things. Everybody wants to hold on to everything and manage by themselves." (Manager2, Priv)

In conclusion, the problems identified are mostly common, but as the interests to solve the problems differ, the window of opportunity in the problem stream opens only partially.

#### Policy stream

When the informants were asked about the possible future changes in the ophthalmology field, not many concrete policy proposals were brought up. The two policy concepts mentioned were the out-sourcing of the services and a model of a public company used in TAUH for hip replacement surgery. However, the first is not a permanent policy alternative as the public sector employs private service providers only in situations of excessive demand. The latter is more a PPP model applied within the public sector and does not represent the concept of PPP as we understand it in this study.

Albeit the PPP did not emerge strongly as a policy proposal, several informants spoke for the cooperation between the public and private sectors, and a clearly negative attitude towards more intense cooperation was expressed only by one of the private sector representatives. Taking this and the literature-based analysis into account it can be said that there are, however, prerequisites for PPP. Through PPP it could be possible to meet the needs of the present in several respects, e.g. the need to solve the disadvantageous situation concerning the suboptimal resource use. Also the structure of dual practice could be challenged as the employers' negotiation power might increase and the dissolution of the specialists' monopoly could become possible.

It must be noticed, however, that the resistance from the employee side may comprise a constraint on PPP. It also seems that the values between the sectors are not shared. Within the public sector a shared ideal of how the services should be produced, i.e. through public provision, prevails, and the actors in this sector are reluctant to turn to the private service providers. At the same time, the big private chain organizations strive for profit and do not regard any change in service production as a fundamental question, unless it has an effect on their market positions.

In conclusion, while there is a lack of proposals for PPP, several prerequisites for it can be found. However, as long as a concrete policy alternative is absent, the window of opportunity cannot be opened fully and probably not even partially. Should the window open fully for PPP, there is a need for a policy entrepreneur to introduce PPP as a solution to the problems. It seems that such an actor is absent at the moment. Hence presently the opportunity window in the policy stream is at least half shut.

#### Political stream

Public opinion is neither strictly for nor against the increasing involvement of private providers in public service provision. Consequently, public opinion and its impact on the possibilities of PPP remain uncertain. Furthermore, the legislation and the public sector's responsibilities also comprise apparent constraints on PPP. However, these constraints do not appear impenetrable, as some solutions to bypass them already exist [4]. In conclusion, the window of opportunity in the political stream opens partially for PPP.

## Discussion

The data set used in this study was relatively small. In a country such as Finland the number of actors relevant to a change as the one discussed in this study is, however, limited. Furthermore, ophthalmology must be regarded as a rather small field of medical expertise. Taking these points into account the data used here represent quite well the relevant scope of actors in the TAUH responsibility area and with some reservations also in the whole of Finland as far as ophthalmology is concerned. In addition to the small data set, it is also crucial to note that the data were primarily collected for use in an innovation management research project mentioned above and hence, PPP was not the original focus of the interviews. It is also possible to identify exogenous factors, such as the current global financial crisis, that may bring some changes to the context of the opportunity window.

As for the study of The Foundation for Municipal Development (2006), we find that it reflects public opinion fairly well also in 2009, three years after the completion of the study, as the changes in the political mood tend to happen slowly. It must be noted, however, that the private sector has traditionally had a relatively large market share in the eye care services compared to the health care services as a whole. Thus, if the views specifically towards private eye care services were asked, public opinion might appear slightly different, i.e. more positive towards the private sector.

The literature-based analysis made it possible to provide a view of the different parties' viewpoints and thus, as an analytical tool, the international literature worked well. Even though the literature concerned different kinds of PPP arrangements in different kinds of contexts, it seems that there might also be some universal factors that affect the initiation of PPP. However, as the analysis was based on the literature, we may have failed to perceive some factors that have an effect on the initiation of PPP in the context of our study.

In the context of PPP it seems that Kingdon's (1995) theory works well when analyzing the stream of problems. The common problems seem to be the most crucial factors when initiating PPP, as without them it is likely that PPP does not appear as a sensible policy solution. Kingdon's (1995) theory was, however, a useful tool when interpreting the results and in the end its role in the study was critical as it made it possible to answer our research question. Even though the theory was originally developed in the context of the US political system, its level of abstraction may be considered universal enough for Western Europe as well. Kingdon's (1995) theory, as well as other theories based on institutionalism, has been used to analyze different kinds of health care reforms in different kinds of health care systems [34]. Taking these considerations into account we thus find the theory suitable for the purpose of the present study. However, research on the applicability of the theory in analyzing health care reforms is called for.

The authors had different roles during the course of the present study. The first author performed the analysis based on the interviews but did not contribute to data collection as did the second author. The interpretation of the analysis was formulated through a dialogue between the authors. Thus, when the reliability of the analysis is concerned, we see the authors' different roles in the study process as complementing each other and hence the analysis as reliable.

## Conclusion

The analysis allows us to conclude that the window of opportunity for PPP opens partially in the field of ophthalmology. However, the question remains: To what extend is the window open, i.e. is the window half-open or half-shut? If we look at the current situation, assuming that in any case some improvements must be made, we find three possible alternatives to solve the situation. On the one hand, the situation can be settled by forming a PPP agreement. On the other hand, the possibilities are either a public monopoly or a fully privatized service system. Even though we found some references to better coordination of work within the public sector, i.e. between primary and secondary health care, a public monopoly does not seem a feasible alternative to solve the situation. This is mainly because the private sector has traditionally been a strong actor in eye health services and because the resources of the public sector are seriously lacking. As for the latter alternative, the informants mentioned that in the future ophthalmology might be a fully privatized specialty of medicine. However, this does not seem likely either, mainly because of the public sector's responsibilities for specialist training and research as well as due to the fact that for the most demanding operations, the necessary resources are available only in the public sector.

If, however, we assume that improvements are not essential, it may be possible that the current situation remains constant. The situation would then appear as path dependent [35]. As shown by our analysis, the situation seems to be disadvantageous for both sectors. Hence, it is likely that both sectors would benefit from a change in the current situation. It is not clear, however, whether the actors fully recognize this fact. It seems that there is a need for a policy entrepreneur, i.e. an actor who is willing to invest his or her time, money and reputation to couple the three streams discussed above [12]. The situation calls for an actor capable of making all the parties see the disadvantages of the current situation as well as the advantages of the policy alternative in question.

There is no doubt that an exogenous pressure, e.g. population ageing, changes in a global or national financial situation or in the market, did not affect the initiation of PPP. However, Kingdon (1995) argues that the absence of a policy entrepreneur leaves the window of opportunity shut, as coupling of the streams may not take place without one. In a situation where prerequisites exist but a concrete policy proposal is missing, a policy entrepreneur may be an even more crucial actor than in a situation with a clear policy alternative. If the policy entrepreneur does appear, the window of opportunity may open fully.

In addition to a policy entrepreneur, the full opening of the window of opportunity calls for the existence of a win-win situation, where both parties gain benefit of some kind. As for the case addressed in this study, the win-win situation must exist particularly at the level of organizational management as in this study we have adopted an organizational viewpoint on PPP. This, however, does not mean that all the actors in the field find PPP favorable. As for the profession of ophthalmologists, recognition of the problem and the interest to solve it seem to be lacking. It may be said that there is a problem, even though differently defined, among all the others but the ophthalmologists. If we studied the same matter from the ophthalmologists' point of view it is likely that any change in the current situation would strike them as negative, as the profession can be seen as one that gains if the current state of affairs prevails. Hence, it is worth noting that the interpretation of the situation discussed here will also depend on the viewpoint adopted.

As the changes in society such as population ageing and technological developments have weakened the possibility of the public sector to meet the needs of the present, there is an increasing need for new health care policies (e.g. PPP) and cooperation between different societal actors in all developed countries. Even though Finnish municipalities and hospital districts can procure services from private service providers, the opportunity is not used to a very large extent [4]. This may be due to the fact that the Nordic countries have had a fairly negative attitude towards the growth of the private health care sector. This can be inferred from the tradition of the Nordic welfare state according to which the responsibility for production of welfare services rests with the public sector. [36] These kinds of ideological dispositions towards the private sector have partly hindered the private sector's involvement in health service provision. However, if considered in the context of ophthalmology, the case is somewhat different, as the services are often produced by private providers. That is to say that in ophthalmology PPP would not necessarily mean a greater market share for the private sector but better possibilities to coordinate service provision as a whole. Thus, the ideological argument against PPP is not necessarily well grounded with regard to ophthalmology.

In conclusion, the time has not yet come for PPP in the context of Finnish ophthalmology services. What the study did reveal, however, was that the discussion on the relationship between the public and private sectors in the context of health care has been put on the agenda. It seems that the previously mentioned co-existence of the public and private sectors seems to be altering towards greater recognition of the other actors operating in the field. Hence, although the time may not be ripe for a partnership at the moment, it seems likely that it might be some time in the future.

## Competing interests

The authors declare that they have no competing interests.

## Authors' contributions

LKT conducted the analysis and most of the literature search. JL contributed to data collection and provided supervision for the first author. Both authors contributed to the methodology, interpretation and concluding remarks.
